# The Ophthalmoscope in Private Practice

**Published:** 1907-04-20

**Authors:** Herbert L. Eason

**Affiliations:** Senior Ophthalmic Surgeon, Guy's Hospital


### SPECIAL ARTICLE.

### THE OPHTHALMOSCOPE IN PRIVATE PRACTICE.

### Its Importance as an Aid to Diagnosis.

By HERBERT L. EASON, M.D., M.S., Senior Ophthalmic Surgeon, Guy's Hospital.

In modern medicine the refinement of the various methods of clinical investigation has reached such a high standard that it is impossible for the busy practitioner to carry out the researches himself, and various successful associations for clinical investigation and research have sprung up to meet the requirements of the time. There are, however, certain instruments, the manipulation of which is comparatively simple, and which should have a place in the armamentarium of the doctor if he is to do the best for his patient and his own credit. Of these ancillary aids to correct diagnosis the ophthalmoscope is undoubtedly the most important. It is little more than fifty years since Helmholtz invented the instrument, but from that date the science of ophthalmology has progressed by leaps and bounds, being at the present time the largest, and possibly the most important, of all the special departments of medicine. By means of the ophthalmoscope the student is enabled to see a small portion of the human economy at work, and from his observations of that minute area which is situated at the back of the eye, to make a shrewd guess at the nature of the morbid processes which are at work all over the body. No other instrument gives the inquirer such accurate information as to the condition of the vascular system, or is of such importance in the diagnosis of growing lesions in the cranial cavity. Yet, to the majority of practitioners, especially those of a preceding generation, the ophthalmoscope is little more than a name, which represents an instrument of appalling difficulty in manipulation, and one which it is almost impossible to learn to use. These apprehensions are, however, unfounded, for with a very small amount of practice the ophthalmoscope may be of the greatest assistance, and by its aid many errors of diagnosis may be avoided, or additional information be obtained which will be of the utmost value to the practitioner in the treatment of his patients. The price of the best ophthalmoscopes is small compared to that of many other instruments which may be required in daily practice, as the best instrument by the best maker can be bought for about three guineas. In addition, for those who cannot master the initial difficulties of the use of the ordinary instrument, which requires some knack to obtain sufficient illumination of the interior of the eye and a good view of the fundus oculi, modern ingenuity has devised forms of self-illuminating ophthalmoscopes which carry their own electric lamp, and throw a beam of light in whichever direction the instrument is pointed. These instruments are now easily obtainable, and with them the merest novice can learn to see the fundus oculi after about an hour's practice. One hint, however, may be given with reference to the use of the ophthalmoscope in private practice; the pupil should always be thoroughly dilated with atropine or some of its quicker, but more expensive, derivatives. With a small pupil it is extremely difficult to make a thorough investigation of the interior of the eye, and some regions, such as the neighbourhood of the macula or yellow spot, it is impossible to see owing to the great constriction of the pupil from the powerful light stimulus. The best light for the purpose is the old-fashioned Argand gas burner, though the ordinary incandescent electric globe answers the purpose fairly well if the glass is frosted.

The ophthalmoscope is required in general medicine for two purposes—in those cases where the patient complains of failure of vision, and it is required that the cause of the failure should be ascertained, and also in that even more important class of case where the patient is suffering from some general constitutional disease where failure of vision, if present at all, is only of secondary importance, but where an examination of the fundus oculi may assist the diagnosis, and may have some bearing upon the treatment or prognosis. In the first class of case it is of importance that the existence of errors of refraction should be ascertained, even though it may not be desired to estimate them accurately, and for this purpose a knowledge of the rudiments of retinoscopy is useful. By means of a retinoscopy it can be ascertained in a few moments whether the patient is suffering from myopia or hypermetropia, and a rough estimate made as to the amount, in order to ascertain whether the amount of refractive error is at all commensurate with the failure of vision.

### An Ophthalmoscopic Examination.

Retinoscopy itself is extremely simple. The patient is seated in a darkened room in front of a light so that the face is in shadow. The investigator stands or sits in front of him about a yard away, a little more rather than less. By means of the ophthalmoscope, a beam of light is cast into the eye, the pupil of which has been previously dilated. The circular aperture of the pupil is then seen surrounding a bright rose-red area, which is caused by the reflection of the beam of light from the vascular choroid. On rotating the mirror from side to side, the circular red area of illumination and the shadow surrounding it are also seen to move from side to side. This effect is most marked if the mirror is moved rapidly, and so that the beam of light travels well from one side of the patient's eye to the other. In some cases it will be seen that the illuminated area and the shadow move in the same direction as the mirror of the ophthalmoscope, that is to say, if the mirror is rotated to the right the shadow will move to the right; in other cases the shadow will move in the opposite direction to the mirror. This movement of the mirror informs the investigator at once as to the nature of the error of refraction, if one be present. In ordinary ophthalmoscopes the mirror is slightly concave, and in cases of hypermetropia the circular area of the illumination on the fundus and its surrounding shadow will move in the opposite direction to the mirror, while in myopia it will move in the same direction. With a plane mirror the movements are reversed. The scope of this article does not permit of a description of the method of estimating the amount of the error, and further details can be obtained from any of the ordinary textbooks. All that it is necessary to point out here is that this investigation as to the existence of any error of refraction should precede any further investigation as to any gross lesion which might cause a failure of vision. In normal eyes the shadow moves against the movement of the mirror, but is not very well marked.

Having ascertained the presence or absence of an error of refraction, the investigator should examine in order the cornea, the lens, and the vitreous. Opacities in each of these will appear as black objects, movable or floating in the vitreous, but fixed in the cornea or lens. Corneal opacities may readily be seen by examination of the eye by oblique illumination with an ordinary lens, as can also superficial opacities of the lens. Having examined the media for opacities, the attention should be directed to the fundus. With a dilated pupil the optic disc will be easily seen by looking into the interior of the eye a little from the outer side on a level with the eye. Any abnormality in the appearance of the disc can then be noted, though it must be remembered that for the purposes of vision the optic disc is the least important part of the eye. That is to say, the optic disc is represented by the well-known blind spot, and any neuritis as a rule only causes some enlargement of that blind area, and not much diminution of vision. A very small patch of choroiditis or a small hæmorrhage, however, if it is placed in the region of the yellow spot will cause a great defect in the patient's central or useful vision, and for this reason great attention should always be paid to this region. It is rather more difficult to see than the optic disc, as there are fewer blood-vessels in that area, and the whole colouring is rather darker and more uniform, and one is apt to be confused by the brilliant reflection of the ophthalmoscope mirror from the anterior surface of the patient's cornea.

Having examined the disc and the macula, attention should then be directed in turn to the upper, lower, inner, and outer quadrants of the eye. Systematic examination of the eye on the lines thus outlined should be carried out in every case of defective vision, and it is surprising how with a little practice it becomes comparatively easy to discover whether or no any opacity of the media of the eye exists, or whether there is any gross lesion in the retina or choroid. It has been mentioned above that this examination should be carried out in all cases where the patient complains of any visual defect, but in general medicine it is of equal, if not of greater, importance that the patient's eyes should be examined as a matter of routine in many common conditions, to take, for example, the condition of persistent headache. Under the stress of modern civilisation and consequent strain upon the entire visual apparatus headaches are becoming increasingly common, and with the greater strain slight defects in the mechanical accuracy of the human eye may lead to such persistent neuralgias as may render life a burden to the patient. Some writers, especially Dr. George Gould, of Philadelphia, have done much good by calling attention to the far-reaching effects of such slight disorders of the visual apparatus, but it is impossible to agree with Dr. Gould in ascribing nearly every disorder to which human flesh is heir to a small degree of refractive error. Every case of headache should, however, be carefully examined with the ophthalmoscope, first of all to ascertain by retinoscopy whether any error of refraction exists, and, secondly, to make oneself quite sure that the headache is not a symptom of some grave intra-cranial lesion.

One of the most appalling mistakes that can be made in practice is by the neglect of such a simple ophthalmoscopic examination to overlook in the case of persistent headache the presence of such inflammation of the optic nerve and its characteristic intra-ocular appearance, to try in turn every empirical remedy for headache with no result, to persist in such treatment for months, and then to learn at the eleventh hour, from the lips of another and more accurate investigator, that the headache is not of a comparatively unimportant sort, but is a symptom of grave intra-cranial pressure caused by a growth. It is no credit to the practitioner, and may be responsible for the early death of the patient, who might have been saved by more prompt treatment at an earlier date.

In routine examinations of the eye as to the possible cause of headaches, a warning may be given as to common conditions which in themselves are not important, but which may be confused with serious intra-cranial symptoms. It is a common mistake with the inexperienced to ascribe the condition of inflammation or neuritis to the disc of a patient suffering from astigmatism or hypermetropia. The classic symptoms of optic neuritis are tortuosity of the vessels with engorgement of the veins, blurring of the edge of the disc with an indistinct margin, and more or less concealment of the vessels by reason of inflammatory exudation. The ordinary hypermetropic fundus shows an optic disc smaller than normal, redder than normal, and for this reason less clearly distinguished from the rest of the fundus. In addition, the vessels may even be slightly more tortuous than normal; and relying on this redness, slight tortuosity of the vessels, and the less distinct margin of the disc, the condition may be labelled neuritis, whereas in reality it is nothing more than a so-called normal abnormality.

In cases of astigmatism also, the blurring and indistinctness of the disc due to the imperfect refraction of the media may be ascribed to neuritis, and mistakes in diagnosis made. As, however, the mistake is a pardonable one, in that a more serious condition is suspected than does in reality exist, it is only necessary to call attention to these small pitfalls to save both patent and practitioner some unnecessary anxiety.

In contrast to the disc in hypermetropia, which may be mistaken for optic neuritis, the myopic disc is often mistaken for atrophy.

One of the most constant appearances in the fundus of the myope is a large disc rather paler than usual, with a crescentic white area, of choroidal atrophy on the outer side, known as the myopic crescent. This appearance generally, especially when seen by the indirect method of examination with the ophthalmoscope, may suggest a partial atrophy of the optic nerve; but to the investigator who can, by means of retinoscopy, quickly ascertain that the eye is myopic, this resemblance will present no difficulties.

### Its Value in Kidney Cases.

The ophthalmoscope is perhaps of most use in general medicine in all stages of nephritis. It is not necessary to emphasise here the frequency with which retinal changes accompany acute and chronic Bright's disease in old and young people, or how valuable is such a diagnostic sign. The condition of the fundus oculi in acute Bright's disease is usually very characteristic. There is some neuritis of the optic disc, and in the region of the yellow spot hæmorrhages and white patches, which are frequently of a shining or glistening white appearance, and these white patches, together with hæmorrhages, are often arranged in radial lines diverging from the yellow spot as a centre. This condition being associated with a certain amount of neuritis, is liable to be mistaken by those who have made no careful examination of the eye with the optic neuritis associated with a cerebral tumour. Patients suffering from granular kidney may have persistent headache and vomiting, and if the eye is only casually examined the presence of some œdema or neuritis of the optic disc may suggest the diagnosis of cerebral tumour; if, however, the characteristic appearance of the fundus in the macular region is also noticed in the course of a thorough examination, the presence of white patches and hæmorrhages will at once rectify the mistake. Though this so-called albuminuric retinitis occurs in both acute and chronic nephritis, it is by far the commonest in the chronic interstitial nephritis of middle age, and in arterio sclerosis. In the middle aged the existence of albuminuric retinitis is of much graver import than in young cases, for as a matter of common experience the prognosis in cases of renal disease associated with albuminuric retinitis is extremely bad. Although it is not strictly true for all cases, it may be taken as roughly accurate that few people live more than two years after the onset of the retinitis. As retinal changes very often cause but slight defect in the patient's vision, the necessity of making a routine examination of the eyes of all patients suffering from renal disease becomes apparent. To be prepared for a grave issue of the case under examination is to be forearmed, and at once the treatment of the patient can be undertaken with much fuller knowledge and confidence than if that additional knowledge had not been obtained. The ophthalmoscopic changes, however, in arterio sclerosis are not limited to the characteristic appearance of albuminuric retinitis. In many cases the first indication that the patient is suffering from some grave vascular defect is obtained from an examination of the fundus oculi.

As has been mentioned above, the choroid and retina being the most vascular tunics in the body, and being observable under high magnification during life, very valuable evidence is afforded of the condition of the vascular system all over the body.

The changes in the retinal arteries in arterio sclerosis are often very characteristic, the vessels becoming rigid, and from the changes in the vessel walls losing their characteristic retinal appearance. The ordinary retinal artery, when seen by the ophthalmoscope, shows as a circular glistening vessel, sometimes pulsating, which has upon its surface one or two bright lines which run longitudinally, and which are the reflection from its cylindrical surface of the light thrown in the eye by the ophthalmoscope. In the normal eye it will also be noticed that where the arteries cross over the veins there is no obstruction to the venous flow. In arterio sclerosis the changes in the vessel wall have altered the appearance of the reflection of the light from the surface of the artery, and they present an appearance similar to that of a metal covered violin string; that is to say, the bright lines running along the surface of the vessel are broken up to some extent, and on account of this change in the appearance the condition is termed that of *gold or silver wire arteries.*

It will also be noticed that the arteries being rigid constrict the veins wherever they cross over them, and in some cases lead to entire obliteration of the veins on the distal side.

The arteries themselves may also be of varying calibre instead of being uniform as in health, and there may be infrequent or numerous hæmorrhages scattered over the whole retina.

Such an ophthalmoscopic picture may be often seen before albumen has been detected in the urine, or before the patient has become aware that he has any serious disease of his kidneys; but, once discovered, the prognosis is very grave, few patients living more than a year or two at the outside.

It is impossible within the limits of such an article as this to mention in detail all the other conditions in which ophthalmoscopic examination of the eye is useful. In many diseases of the central nervous system, as locomotor ataxy or disseminated sclerosis, the ophthalmoscope may help to confirm an opinion already half formed; and in many diseases of the blood, such as leukæmia, corroborative evidence may be found in the condition of the fundus oculi.

Diabetes also, tubercular meningitis, and other similar conditions may give rise to characteristic appearances of the fundus; and in such grave conditions as melanotic sarcoma of the choroid early discovery of the malignant centre may lead to a timely operation, and the saving of the patient's life.

This outline of some of the more common conditions in which the ophthalmoscope is of inestimable value may, it is hoped, stimulate practitioners to use this valuable instrument more freely, for it can be confidently predicted that habitual use will only serve to emphasise its ever-increasing value as an aid to diagnosis.

